# Determination of the postprandial cut-off value of triglyceride after a daily meal corresponding to fasting optimal triglyceride level in Chinese subjects

**DOI:** 10.3389/fnut.2023.1037270

**Published:** 2023-03-02

**Authors:** Yingying Xie, Liling Guo, Hao Chen, Jin Xu, Peiliu Qu, Liyuan Zhu, Yangrong Tan, Miao Zhang, Tie Wen, Ling Liu

**Affiliations:** ^1^Department of Cardiovascular Medicine, The Second Xiangya Hospital, Central South University, Changsha, Hunan, China; ^2^Research Institute of Blood Lipid and Atherosclerosis, Central South University, Changsha, Hunan, China; ^3^Modern Cardiovascular Disease Clinical Technology Research Center of Hunan Province, Changsha, Hunan, China; ^4^Cardiovascular Disease Research Center of Hunan Province, Changsha, Hunan, China; ^5^Department of Emergency Medicine, The Second Xiangya Hospital, Central South University, Changsha, China; ^6^Emergency Medicine and Difficult Diseases Institute, The Second Xiangya Hospital, Central South University, Changsha, China

**Keywords:** triglyceride, remnant cholesterol, postprandial, cut-off value, daily meal, optimal

## Abstract

**Background:**

According to the 2021 consensus statement about triglyceride (TG)-rich lipoproteins and their remnants from the European Atherosclerosis Society (EAS), fasting TG level < 1.2 mmol/L is regarded as optimal, otherwise considered as non-optimal TG (NoTG). However, the postprandial cut-off value after a daily meal corresponding to a fasting TG level of 1.2 mmol/L has not been explored.

**Materials and methods:**

Six hundred and eighteen inpatients aged 18 to 70 were recruited in this study. Among them, 219 subjects had fasting TG levels < 1.2 mmol/L (i.e., OTG group), and 399 subjects had fasting TG levels ≥ 1.2 mmol/L (i.e., NoTG group). Serum levels of blood lipids, including calculated non-high-density lipoprotein cholesterol (non-HDL-C) and remnant cholesterol (RC), were monitored at 0, 2, and 4 h after a daily Chinese breakfast according to their dietary habits. Receiver operating characteristic (ROC) curve analysis was used to determine the postprandial cut-off value corresponding to the fasting TG level of 1.2 mmol/L. Kappa statistics were performed to determine the consistency between fasting and postprandial cut-off values in determining whether TG was optimal. Univariate and multivariate logistic regression analyses were conducted to evaluate the associations between NoTG and potential confounders. Subgroup analyses were performed to explore the association between postprandial TG levels at 4h (pTG4h) and NoTG in greater detail.

**Results:**

Postprandial levels of TG and RC significantly elevated and peaked at 4h after a daily breakfast in two groups (*P* < 0.05). The optimal cut-off value at 4h corresponding to fasting TG of 1.2 mmol/L was 1.56 mmol/L. According to the fasting cut-off value, the percentage of patients with NoTG was 64.6% in the fasting state while increasing obviously to 73.3–78.4% at 2 and 4h, respectively, after a daily Chinese breakfast. According to the postprandial cut-off value, the percentage of patients with NoTG at 4h after a daily Chinese breakfast was 62.6% which was close to 64.6% in the fasting state. The Kappa coefficient was 0.551, indicating a moderate consistency between the fasting and postprandial cut-off values in the diagnosis of NoTG. Moreover, the subjects with NoTG determined by the postprandial TG cut-off value had an obviously higher postprandial level of RC (1.2 vs. 0.8 mmol/L) and percentage of HRC (37.1 vs. 32.1%) than those determined by the fasting TG cut-off value. Multivariate logistic regression analyses demonstrated that except for BMI, pTG4h emerged as an independent predictor of not. Subgroup analyses revealed that the association between pTG4h and NoTG was consistent across subgroups.

**Conclusion:**

Taken together, we for the first time determined TG 1.56 mmol/L as the postprandial cut-off value corresponding to fasting TG 1.2 mmol/L in Chinese subjects. This could make it more convenient to determine whether TG is optimal or not in the fasting or postprandial state.

## Introduction

The elevated level of low-density lipoprotein cholesterol (LDL-C) is regarded as an independent risk factor for atherosclerotic cardiovascular disease (ASCVD). Controlling low-density lipoprotein cholesterol (LDL-C) to the target level is the primary goal for patients with ASCVD to reduce clinical cardiovascular events (1). Different from fasting LDL-C level, the relationship between fasting triglyceride (TG) level and ASCVD was controversial (2). Since people are in a postprandial (i.e., non-fasting) state most of the day, more attention is focused on the relationship between postprandial blood lipids and ASCVD.

Strong evidence supports that elevated postprandial levels of triglyceride, as well as LDL-C, can independently predict the risk of ischemic heart disease, including that of myocardial infarction (3). Based on these new findings, the testing of postprandial blood lipids has been recommended in routine clinical practice in Europe since 2016 (4). The 2019 ESC/EAS guideline for the management of dyslipidemias classifies fasting TG (fTG) of < 1.7 mmol/L (150 mg/dL) as desirable, noting that fTG ≥ 1.7 mmol/L (150 mg/dL) is associated with the increased risk of ASCVD (5). Moreover, controlling non-high-density-lipoprotein cholesterol (non-HDL-C) to the target level is the secondary goal to reduce the risk of ASCVD. Non-HDL-C is the total amount of cholesterol contained in lipoproteins other than high-density lipoprotein (HDL), which includes not only LDL-C but also cholesterol in other atherogenic lipoproteins, such as TG-rich lipoproteins (TRLs) and their hydrolyzed products, i.e., remnant lipoproteins (6). The cholesterol in remnant lipoproteins (i.e., remnant cholesterol, RC) is an important part of non-HDL-C, especially in patients with hypertriglyceridemia. Hypertriglyceridemia represents the increased number of remnant lipoproteins in circulation. Compared with nascent TRLs, remnant lipoproteins contain more cholesterol ester, have smaller diameters, and thus are regarded as atherogenic as LDL.

Hypertriglyceridemia has been defined as fTG levels of 1.7 mmol/L (150 mg/dL) or higher (5, 7). For example, the 2018 ACC/AHA guideline classifies moderate hypertriglyceridemia as 1.7–5.59 mmol/L (150–499 mg/dL) and severe hypertriglyceridemia as 5.6 mmol/L (500 mg/dL) or more (7). The 2016 Chinese guideline classifies appropriate fTG level as < 1.7 mmol/L (150 mg/dL), borderline hypertriglyceridemia as 1.7–2.29 mmol/L (150–199 mg/dL) and hypertriglyceridemia as ≥ 2.3 mmol/L (200 mg/dL) (8). The prevalence of hypertriglyceridemia is 16.9% (8), supporting that hypertriglyceridemia is the most common form of dyslipidemia in the Chinese population. Recently, the definition of TG elevation has been updated again (9). According to the 2021 consensus statement about TRLs and their remnants from the EAS, optimal TG level is defined as fasting TG < 1.2 mmol/L (100 mg/dL), borderline elevation as 1.2 mmol/L ≤ fTG < 1.7 mmol/L and elevation as fTG ≥ 1.7 mmol/L (150 mg/dL). To recommend postprandial blood lipids testing in routine clinical practice, the 2016 consensus statement from ESC proposes that the cut-off values for high TG and high RC (HRC) in the fasting state are 1.7 and 0.8 mmol/L, respectively, and those in the postprandial state are 2.0 and 0.9 mmol/L, respectively (4). However, there was no idea about the postprandial cut-off value of TG after a daily meal corresponding to the fasting optimal TG cut-off value of 1.2 mmol/L.

This study aimed to determine the postprandial cut-off value after a daily meal corresponding to fTG level of 1.2 mmol/L in Chinese subjects, and to compare the roles of fasting and postprandial cut-off values in determining TG is optimal or not.

## Materials and methods

### Study subjects

Six hundred and eighteen inpatients of Chinese Han nationality aged 18 to 70 were enrolled in this study in the Department of Cardiovascular Medicine of the Second Xiangya Hospital from March 2017 to July 2020. Among them, 219 patients had optimal TG (OTG group: fTG < 1.2 mmol/L) and 399 patients had non-optimal TG (NoTG group: fTG ≥ 1.2 mmol/L). All of them were excluded from a history of digestive disease, autoimmune disease, hepatic and renal diseases, mental diseases, cancer or other severe medical diseases, or NYHA heart function class III-IV before getting involved. This study was approved by the Ethics Committee of the Second Xiangya Hospital of Central South University and informed consent was obtained from all participants.

### Specimen collection

After at least 8 h of overnight fasting, all subjects finished breakfast according to their own dietary habits within 15–20 min. The traditional Chinese breakfast that most Chinese people are used to usually includes the following categories: Meal 1 mainly included soybean milk, and fried dough sticks, which contain about 200–300 kcal. Meal 2 mainly included milk, bread, or eggs, which contain about 170–250 kcal. Meal 3 mainly included noodles or porridge, which contains about 220–270 kcal. Meal 4 mainly included steamed buns or rolls, which contain about 250–310 kcal. There is little difference in calories among different types of Chinese breakfast. All individuals were asked about their habitual dietary types and subjects who preferred one of the above types of breakfasts were included. It is not recommended that the subjects smoke, drink wine or beer, eat any food, or do strenuous exercise within 4h after breakfast, except for a little water and a slow walk. Venous blood samples were collected at fasting state, 2 and 4h after breakfast.

### Laboratory assays

The serum was separated from the venous blood samples. Serum levels of TG and total cholesterol (TC) were measured by automated enzymatic assays, and those of LDL-C and high-density lipoprotein cholesterol (HDL-C) were determined by the direct method, and were measured by laboratory technicians who are unaware of this study through a HITACHI 7170A analyzer (Instrument Hitachi Ltd., Tokyo, Japan) The detection kits were provided by Japan and Pure Pharmaceutical Industry Co., (Wako). Levels of RC and non-HDL-C were estimated by the following formulas, RC = TC–(HDL-C)–(LDL-C) and non-HDL-C = TC–(HDL-C) (10), respectively.

### Estimated sample size

The sample size was estimated based on the following calculation formula: n_1_ = n_2_ = 2 × [(t_α_ + t_β_)s/δ]^2^ (n_1_ and n_2_ are the required contents of the two samples respectively; t_α_ and t_β_ are the *t* values corresponding to inspection level α and type II error probability β, respectively; s is the estimated value of the overall standard deviation; δ is the difference between the two means). Using a two-sample *t*-test, the test efficiency is 90% at the test level of 5% on both sides. The required estimated value of sample size is calculated based on the pre-experimental data of 2 and 4h after a meal, and the larger value is taken as the sample size required for this study. According to the difference between NoTG groups at 2 and 4h after a meal, the calculated sample size is 18 and 45, respectively. Taking the maximum value of each group, about 45 people are needed in each group, and a total of 90 people need to be included in this study. This study included 618 individuals, 219 in the OTG group and 399 in NoTG group. So, the sample size is appropriate for this present study, and sample size estimation was performed as further validation after the study design.

### Statistical analysis

Quantitative variables of normal distribution were expressed as mean ± standard deviation (SD), and Qualitative variables were expressed as numbers and percentages. One-way analysis of variance (ANOVA) for repeated measures was used to evaluate the difference between the mean values of variables within one group. ANOVA for completely randomized measures was used to evaluate the difference between the mean values of variables between the two groups. Qualitative variables were compared using the Chi-square test for R x C. The area under the curve (AUC) and AUC increment (iAUC) that represent the increase in the area after a daily meal relative to the fasting level of TG and RC were estimated by the trapezoid method. Receiver operating characteristic (ROC) curve analysis was used to determine the postprandial cut-off value corresponding to the fasting TG level of 1.2 mmol/L. Reliability analysis was performed with the Kappa statistic to determine the consistency between fasting and postprandial cut-off values in determining whether TG was optimal. Univariate and multivariate logistic regression analysis was conducted to examine associations between NoTG and several potential confounders including age, gender, BMI, current smoking, history of hypertension, DM and CHD. Subgroup analyses were performed after stratification by age (< 55 or ≥ 55 years), gender (male or female), BMI (< 24 or ≥ 24 kg/m^2^), current smoking (yes or no), history of hypertension (yes or no), DM (yes or no) and CHD (yes or no) to identify any modification caused by these variables. All statistical analyses were performed with SPSS version 26.0. All *P* values were 2-tailed, and *P* < 0.05 was considered statistically significant.

## Results

### Clinical characteristics and fasting blood lipids of two groups

There were 618 participants aged 18 to 70 recruited in this study population, and fasting NoTG (TG ≥ 1.2 mmol/L) was found in 399 (64.6%) of the individuals. The general characteristics of the OTG group (*n* = 219) and the NoTG group (*n* = 399) were presented in Table 1. There was no significant difference in age, gender, systolic or diastolic blood pressure, heart rate, the percentages of smoking and recent use of lipid-lowering drugs between the two groups. Body mass index in the NoTG group was significantly higher than that in the OTG group (*P* < 0.05). The proportion of overweight and obese individuals and that of patients with CHD or diabetes mellitus in the NoTG group were significantly higher than those in the OTG group (*P* < 0.05).

**TABLE 1:** Comparison of clinical features between two groups.

	OTG (*n* = 219)	NoTG (*n* = 399)	*P*-value
Age (year, SD)	55.3 ± 12.6	53.9 ± 10.0	NS
Men, n (%)	140 (64.0)	275 (68.9)	NS
BMI, kg/m^2^	23.4 ± 3.2	25.3 ± 3.6	<0.0001
OWand OB, n (%)	85 (38.8)	260 (65.2)	<0.0001
Current smoking, n (%)	79 (36.1)	171 (43.1)	NS
Systolic pressure (mmHg)	130.0 ± 19.1	132.0 ± 20.0	NS
Diastolic pressure (mmHg)	80.2 ± 13.5	82.0 ± 12.2	NS
Heart rate (bpm)	78.7 ± 17.1	78.5 ± 15.0	NS
DM, n (%)	31 (14.2)	83 (20.9)	<0.05
CHD, n (%)	101 (46.1)	217 (54.4)	<0.05
Taking statins, n (%)	78 (35.6)	165 (41.8)	NS
Taking statins ≥ 3 months, n (%)	37 (16.9)	71 (18)	NS
Ezetimibe, n (%)	1 (1.2)	2 (1.4)	NS
TC, mmol/L	3.78 ± 0.80	4.12 ± 0.77	<0.0001
LDL-C, mmol/L	2.20 ± 0.68	2.62 ± 0.67	<0.0001
HDL-C, mmol/L	1.17 ± 0.30	1.0 ± 0.21	<0.0001
Non-HDL-C, mmol/L	2.60 ± 0.70	3.14 ± 0.70	<0.0001
TG, mmol/L	0.90 ± 0.21	2.06 ± 0.78	<0.0001
RC, mmol/L	0.37 ± 0.19	0.51 ± 0.18	<0.0001

NoTG group, fasting TG ≥ 1.2 mmol/L; OTG group, fasting TG < 1.2 mmol/L; BMI, body mass index; OW and OB, overweigh (BMI 24–27.9) and obesity (BMI ≥ 28); DM, diabetes mellitus; CHD, coronary heart disease; TC, total cholesterol; HDL-C, high-density lipoprotein cholesterol; LDL-C, low-density lipoprotein cholesterol; non-HDL-C, non-high-density lipoprotein cholesterol; TG, triglyceride; RC, remnant lipoprotein cholesterol.

### Postprandial changes of blood lipids in two groups

In the fasting state, levels of TG, TC, LDL-C, non-HDL-C, and RC were significantly higher while fasting HDL-C level was markedly lower in the NoTG group (*P* < 0.05, Table 1).

**FIGURE 1:**
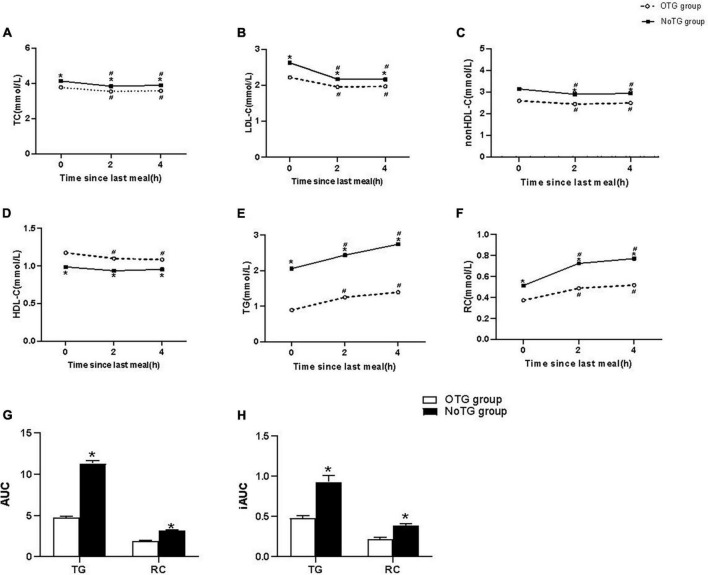
Postprandial changes in blood lipids after a daily meal in two groups. **(A,B,D,E)** The changes in serum concentrations of total cholesterol (TC), low-density lipoprotein cholesterol (LDL-C), high-density lipoprotein cholesterol (HDL-C), and triglyceride (TG). **(C,F)** The changes in serum concentrations of non-HDL-C and remnant cholesterol (RC) were determined by calculated methods. **(G)** Comparison of the total area under the curve (AUC) for TG and RC between two groups. **(H)** Comparison of increase in AUC (iAUC) for TG and RC between two groups. Optimal triglyceride (OTG) group: fasting TG < 1.2 mmol/L. Non-optimal triglyceride (NoTG) group: fasting TG ≥ 1.2 mmol/L. Values are mean ± standard error (SE). One-way analysis of variance (ANOVA) for repeated measures within a group or ANOVA for completely randomized measures between groups was used to assess any differences between the means of the variables, as appropriate. *P < 0.05 when compared with the patients in the OTG group. ^#^*P* < 0.05 when compared with the fasting level in the same group.

After a daily breakfast, levels of TG and RC significantly increased, while those of TC, LDL-C and non-HDL-C significantly decreased in the two groups (*P* < 0.05). Both TG and RC levels peaked at 4h after a daily breakfast in both groups. Postprandial levels of TC, LDL-C, non-HDL-C, TG, and RC at 2 and 4h after a daily meal in the NoTG group were significantly higher than those in the OTG group (*P* < 0.05, Figures 1A–F).

Both AUC and iAUC of TG or RC levels in the NoTG group were significantly higher than those in the OTG group (*P* < 0.05, Figures 1G, H).

### Evaluation of NoTG according to the new postprandial cut-off value

**FIGURE 2:**
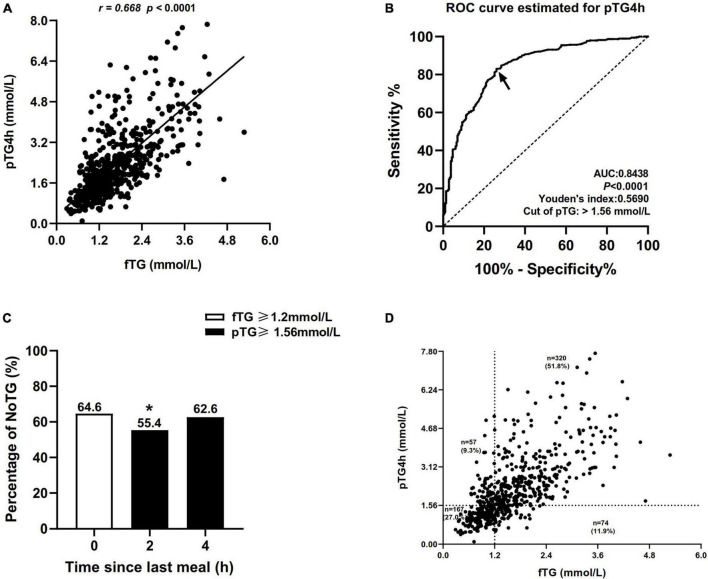
Comparisons of the percentages of non-optimal triglyceride (NoTG) at different time-points. **(A)** Correlation between the level of fasting TG (fTG) and pTG4h. **(B)** Receiver operating characteristic (ROC) analysis and Youden’s index determined a cut-off value for postprandial TG level at 4h (pTG4h) after a daily meal; the cut-off value was indicated by a solid arrow. **(C)** Comparisons of the percentages of NoTG at different time-points according to the different cut-off values. **(D)** Distribution of the levels of fTG and pTG4h in all subjects. *P < 0.05 when compared with the fasting state. Optimal triglyceride (OTG) group: fasting TG < 1.2 mmol/L. Non-optimal triglyceride (NoTG) group: fasting TG ≥ 1.2 mmol/L. Values are represented as n (%) as appropriate.

Correlation analysis showed that the correlation between fTG and postprandial TG level at 4h (pTG4h) was the strongest (*r* = 0.668, *P* < 0.0001, Figure 2A), followed by that between fTG and total postprandial TG (postprandial TG at 2 and 4h) (*r* = 0.632, *P* < 0.0001, Supplementary Figure 1A), and then by that between fTG and postprandial TG level at 2 h (pTG2h) (*r* = 0.626, *P* < 0.0001, Supplementary Figure 1C).

Receiver operating characteristic (ROC) curve analysis showed that the optimal cut-off value for TG at 4h corresponding to fasting TG 1.2 mmol/L was 1.56 mmol/L (sensitivity 83%, specificity 74%, and AUC 0.8438, Figure 2B).

According to the fasting cut-off value, the percentage of patients with NoTG was 64.60% at the fasting state while increasing obviously to 73.3–78.4% at 2 and 4h, respectively, after a daily Chinese breakfast (Supplementary Figure 2). According to the postprandial cut-off value, the percentage of patients with NoTG at 4h after a daily Chinese breakfast was 62.6% which was close to that in the fasting state (Figure 2C).

Subsequently, we evaluated the distribution of the fTG and pTG4h according to the fasting and postprandial cut-off values in all subjects. The number of individuals who were double-high (i.e., fTG ≥ 1.2 mmol/L and pTG 4h ≥ 1.56 mmol/L) reached 320 in 6l8, accounting for 51.8%. The number of individuals who were double-optimal (i.e., fTG < 1.2 mmol/L and pTG 4h < 1.56 mmol/L) was 167 in 618, accounting for 27.0%. Some patients with fasting TG levels ≥ 1.2 mmol/L showed optimal postprandial TG levels (74, 11.9%), while others with fasting TG levels < 1.2 mmol/L were found with non-optimal postprandial TG levels (57, 9.3%) (Figure 2D).

Kappa analysis showed that the Kappa coefficient was 0.551, indicating a moderate consistency between the fasting and postprandial cut-off values in the diagnosis of NoTG.

### Comparison of the distribution of NoTG and HRC according to different cut-off values

**FIGURE 3:**
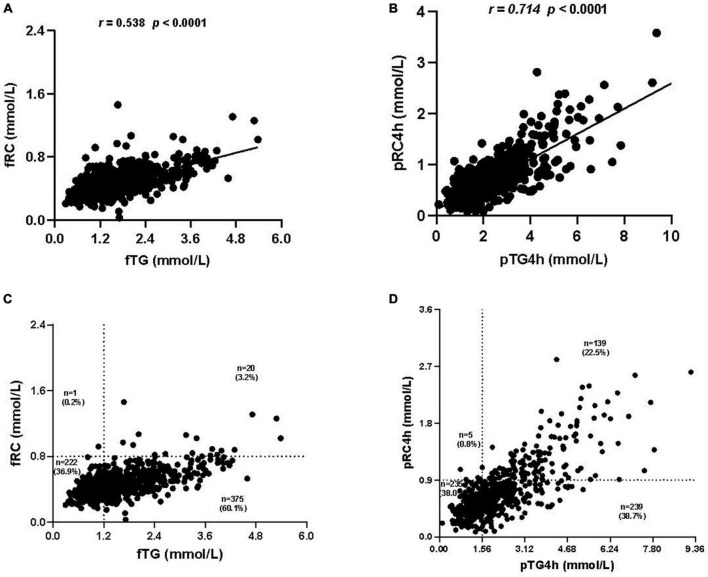
Correlation analysis between the levels of triglyceride (TG) and remnant cholesterol (RC). **(A)** Correlation between fasting TG (fTG) and fasting RC (fRC) levels. **(B)** Correlation between postprandial TG level at 4h (pTG4h) and postprandial RC level at 4h (pRC4h). **(C)** Distribution of the fTG and fRC levels in all subjects. **(D)** Distribution of the pTG4h and pRC4h levels in all subjects.

Correlation analysis between levels of TG and RC showed that the correlation between pTG4h and postprandial RC level at 4h (pRC4h) (*r* = 0.714, *P* < 0.0001) was stronger than that between fTG and fasting RC (fRC) (*r* = 0.538, *P* < 0.0001) (Figures 3A, B) and that between pTG2h and pRC2h (*r* = 0.525, *P* < 0.0001, Supplementary Figure 1B), then by that pTG2&4h and pRC2&4h (*r* = 0.392, *P* < 0.0001, Supplementary Figure 1D).

The distribution of TG and RC was observed in both fasting and postprandial states. Notably, the number of individuals who were double-high in the fasting state (i.e., fTG ≥ 1.2 mmol/L and fRC ≥ 0.8 mmol/L) was only 20 in 618, accounting for 3.2% (Figure 3C), while that in the postprandial state (i.e., pTG4h ≥ 1.56 mmol/L and pRC4h ≥ 0.9 mmol/L) reached 139 in 618, accounting for 22.5% (Figure 3D). The number and proportion of individuals who were double-optimal in the fasting state (i.e., fTG < 1.2 mmol/L and fRC < 0.8 mmol/L) and postprandial state (i.e., pTG4h < 1.56 mmol/L and pTG4h < 0.9 mmol/L) was similar, i.e., 222 in 6l8 (36.9%) vs. 235 in 618 (38.0%) (Figures 3C, D).

### Comparison of RC level and the percentage of HRC according to different cut-off values of TG

**FIGURE 4:**
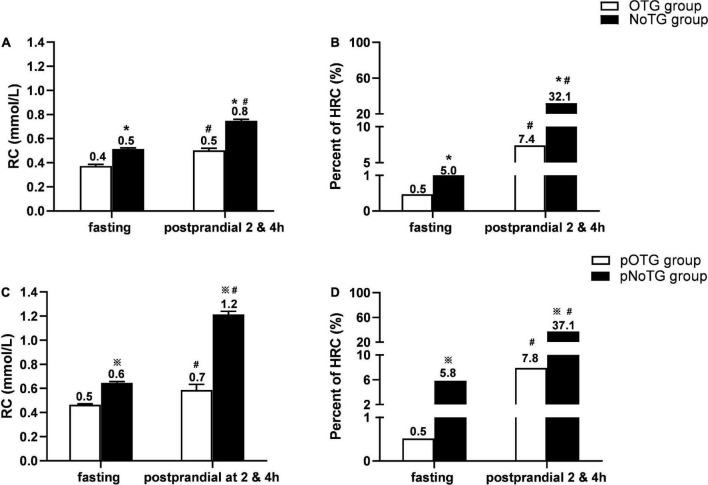
Comparison of RC levels and percentages of HRC according to different cut-off values. **(A)** Comparison of levels of RC and percentages of high RC (HRC) between the OTG group (*n* = 219) and NoTG group (*n* = 399) classified by the fasting cut-off value of TG 1.2 mmol/L. **(B)** Comparison of percentages of high RC (HRC) between the OTG group (*n* = 219) and NoTG group (*n* = 399) classified by the fasting cut-off value of TG 1.2 mmol/L. **(C)** Comparison of levels of RC and percentage of HRC between the pOTG group (*n* = 231) and pNoTG group (*n* = 387) classified by the postprandial cut-off value of TG 1.56 mmol/L. **(D)** Comparison of percentages of HRC between the pOTG group (*n* = 231) and pNoTG group (*n* = 387) classified by the postprandial cut-off value of TG 1.56 mmol/L. Optimal triglyceride (OTG) group: fasting TG < 1.2 mmol/L. Non-optimal triglyceride (NoTG) group: fasting TG = 1.2 mmol/L. Postprandial optimal TG (pOTG, i.e. pTG4h < 1.56 mmol/L), and postprandial non-optimal TG (pNoTG, i.e. pTG4h = 1.56 mmol/L). *P < 0.05 when compared with the OTG group. ^#^*P* < 0.05 when compared with the fasting state within the same group. ^
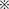
^*P* < 0.05 when compared with the pOTG group. Values are mean ± standard error (SE) or n (%) as appropriate.

Compared with the OTG group, the NoTG group had significantly higher fasting and postprandial RC levels as well as the percentage of fasting HRC (fasting RC, i.e., fRC, ≥ 0.8 mmol/L) (Figures 4A, B). According to the postprandial cut-off value of optimal TG (i.e., 1.56 mmol/L), all subjects were divided into patients with postprandial OTG (pOTG: pTG4h < 1.56 mmol/L) and those with postprandial NoTG (pNoTG: pTG4h ≥ 1.56 mmol/L). Similarly, patients with pNoTG also had significantly higher postprandial RC levels as well as the percentage of postprandial HRC (postprandial RC, i.e., pRC ≥ 0.9 mmol/L) than those with pOTG (Figures 4C, D).

More importantly, patients with pNoTG showed higher postprandial RC levels (1.2 vs. 0.8 mmol/L) and the percentage of postprandial HRC (37.1 vs. 32.1%) than those with fasting NoTG (fTG ≥ 1.2 mmol/L), although their fasting RC levels and HRC percentages seemed similar (Figure 4B).

### Logistic regression and subgroup analyses

**FIGURE 5:**
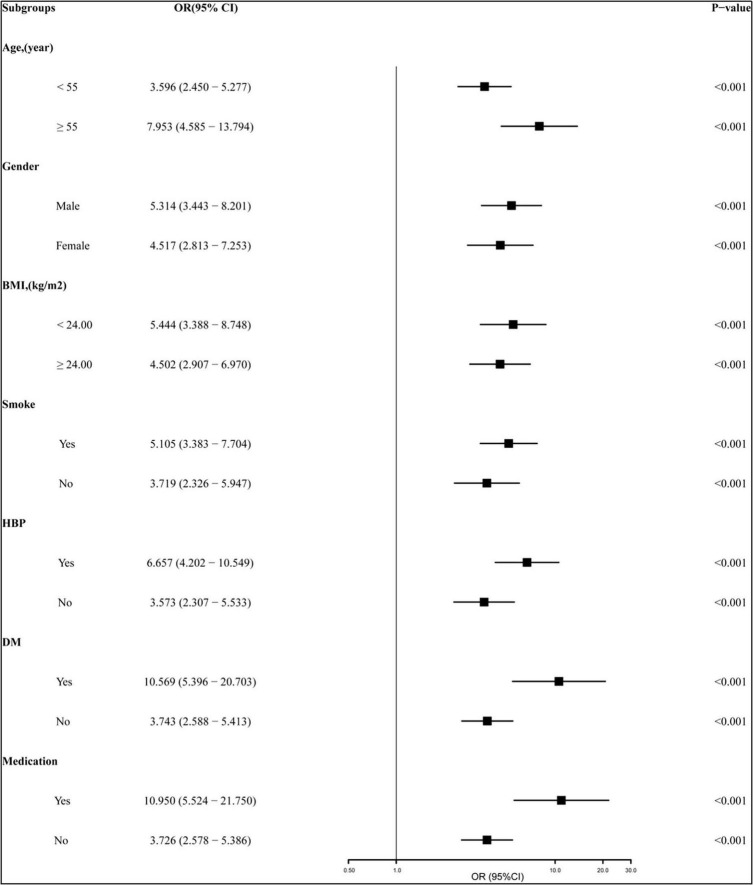
Subgroup analyses of the relationship between pTG4h and non-optimal TG (NoTG). The study population was stratified by age (< 55 years vs. ≥ 55 years), gender (male vs. female), BMI (< 24 kg/m^2^ vs. ≥ 24 kg/m^2^), current smoking (yes vs. no), hypertension (yes vs. no), DM (yes vs. no), and medication (yes vs. no). BMI, body mass index; DM, diabetes mellitus; CHD, coronary heart disease; OR, odds ratio; CI, confidence interval; pTG4h, postprandial triglyceride level at 4 h; NoTG, non-optimal triglyceride.

To investigate the association between all variables and NoTG, univariate and multivariate logistic regression analyses were conducted (Table 2). Univariate logistic regression analyses showed that age [odds ratio (OR): 1.008 (95% CI: 0.993–1.023); *P* = 0.325], being male [OR: 1.996 (95% CI: 1.402–2.766); *P* < 0.001], BMI [OR: 1.174 (95% CI: 1.113–1.239); *P* < 0.001], current smoking [OR: 1.341 (95% CI: 0.954–1.884); *P* = 0.091], history of hypertension [OR: 1.269 (95% CI: 0.906–1.778); *P* = 0.166], DM [OR: 1.593 (95% CI: 1.015–2.499); *P* = 0.043], CHD [OR: 1.393 (95% CI: 1.001–1.939); *P* = 0.049], medication [OR: 1.262 (95% CI: 0.900–1.771); *P* = 0.177], and pTG4h [OR: 5.133 (95% CI: 3.765–6.996); *P* < 0.001] were all significantly associated with NoTG. Subsequent multivariate regression analysis revealed that except for BMI [OR: 5.133 (95% CI: 3.765–6.996); *P* < 0.001], only pTG4h [OR: 4.490 (95% CI: 3.590–6.802); *P* < 0.001] was found to be an independent predictor of NoTG. Additionally, we performed subgroup analyses stratified by age, gender, BMI, smoke, hypertension, DM and medication to explore the association between pTG4h and NoTG in greater detail. It was found that the association of pTG4h and NoTG was consistent across subgroups (Figure 5).

**TABLE 2:** Univariate and multivariate logistic regression analyses.

Variables	Univariate logistics regression			Multivariate logistics regression		
	**OR**	**CI (95%)**	***P*-value**	**OR**	**CI (95%)**	***P*-value**
Age (year)	1.008	0.993–1.023	0.325	1.016	0.994–1.038	0.163
Male	1.996	1.402–2.766	<0.001	1.329	0.996–1.344	0.065
BMI (kg/m^2^)	1.174	1.113–1.239	<0.001	1.107	1.042–1.176	0.001
Current smoking	1.341	0.954–1.884	0.091	0.872	0.513–1.482	0.613
Hypertension	1.269	0.906–1.778	0.166	0.994	0.825–1.198	0.950
DM	1.593	1.015–2.499	0.043	1.071	0.627–1.828	0.803
CHD	1.393	1.001–1.939	0.049	1.119	0.624–1.945	0.739
Medication	1.262	0.900–1.771	0.177	1.101	0.595–1.677	0.998
pTG4h	5.133	3.765–6.996	<0.001	4.942	3.590–6.802	<0.001

OR, odds ratio; BMI, body mass index; DM, diabetes mellitus; CHD, coronary heart disease; pTG4h, postprandial triglyceride levels at 4h.

## Discussion

In this study, significant postprandial hyperlipidemia mainly characterized by elevated TG and RC levels after a daily meal was observed in patients with NoTG, particularly at 4h. What’s more, There were higher AUC and iAUC of TG and RC in the NoTG group than in the OTG group, which indicated the more intensive and enduring postprandial reactive increases in TG and RC in those with fasting non-optimal TG levels. Through ROC curve analysis, the cut-off value of postprandial optimal TG level in Chinese subjects was first determined as 1.56 mmol/L, which corresponded to the fasting one recommended by the 2021 ESA consensus statement, i.e., 1.2 mmol/L (9). And there was a moderate agreement between fasting and postprandial cut-off values in the diagnosis of optimal TG level with a Kappa coefficient of 0.551. Multivariate logistic regression analyses demonstrated that pTG4h was an independent predictor of NoTG, and subgroup analyses revealed the association between pTG4h and NoTG was consistent across subgroups. Hence, this could make it more convenient to determine whether TG is optimal or not, either in the fasting state or after a daily meal.

The postprandial state is a critical period in the progress of atherosclerosis (11, 12). Two large population-based studies, the CORonary Diet Intervention with Olive Oil and Cardiovascular PREVention (CORDIOPREV) study (13) and the Genetics of Lipid Lowering Drugs and Diet Network (GOLDN) study (14) have proposed that an oral-fat tolerance test (OFTT) in clinical practice can be conducted to identify postprandial hyperlipidemia in subjects with fasting TG between 1 and 2 mmol/L (89–180 mg/dL) because approximately half of them have hidden postprandial hyperlipidemia, which may influence treatment (15). And there were 318 subjects (about 51.50%) diagnosed with coronary heart disease. However, given the current popularization of healthy diet education, a high-fat diet is unacceptable, especially for patients with ASCVD. Hence, the subjects are more likely to accept the daily breakfast according to their own dietary habits, other than a high-fat diet. Additionally, there is no unified standard high-fat meal scheme in the clinic, even though OFTT has received a lot of attention and recommendations recently (13–15). More importantly, the 2016 European expert consensus recommended that blood lipids should be detected after daily meals rather than standard high-fat meals (4). This is one of the main reasons why we paid more attention to postprandial levels of blood lipids after a daily meal.

Emerging studies have recommended that 4h after an OFTT was the most representative time-point to measure TG concentrations owing to the biggest difference between TG levels at 4h after a high-fat diet and that in a fasting state (16). Similarly, we found that TG levels reached a peak at 4h in two groups after a daily breakfast. More importantly, the correlation between fasting and postprandial TG levels was the strongest at 4h after a daily breakfast. That’s why we chose 4h after a daily meal as the time-point to evaluate the cut-off value to determine the postprandial optimal TG cut-off value.

According to the new postprandial cut-off value determined by ROC curve analysis, the percentage of NoTG at 4h was close to that in the fasting state according to the cut-off value of TG 1.2 mmol/L recommended by the 2021 EAS consensus statement. The number of patients with fasting TG ≥ 1.2 mmol/L and postprandial TG ≥ 1.56 mmol/L as well as fasting TG < 1.2 mmol/L and postprandial TG < 1.56 mmol/L reached 489 in 618, accounting for about 80% in this study. It suggested that there was at least moderate coincidence and consistency in the determination of optimal TG between the two cut-off values. Moreover, it indicates that in addition to patients who are in a fasting state, those who visit their doctors after meals can also obtain information on whether their TG levels are optimal or not.

It has been known that increased RC level can independently predict the residual risk of ASCVD (17). Although TG level is closely associated with RC level, high RC is a more direct risk factor for atherosclerosis compared with high TG because remnant lipoproteins can enter the subendothelial area of the artery and promote the formation of foam cells (18). It is recommended by the 2016 consensus statement from ESC that RC levels should not exceed 0.8 mmol/L in the fasting state and 0.9 mmol/L in the postprandial state (4), otherwise, it may indicate an increased risk of atherosclerosis. In this study, more subjects with postprandial HRC were found in those without optimal TG levels according to the postprandial cut-off value than those according to the fasting cut-off value. It supports that postprandial TG increase could expose the artery to more remnant lipoproteins in circulation. Hence, the postprandial cut-off value to determine whether TG is optimal or not could be conducive to improving the detection rate of HRC patients and promoting timely lifestyle intervention.

Notably, some patients with fasting TG levels ≥ 1.2 mmol/L had optimal postprandial TG levels (74, 11.9%), which may be due to individual differences in postprandial TG metabolism and/or dietary habits. However, other patients with fasting TG levels < 1.2 mmol/L were found with NoTG in the postprandial state (57, 9.0%), which indicated that a considerable number of subjects with NoTG will be omitted if postprandial blood lipids were not evaluated. As we all know, some patients with diabetes only show elevated postprandial blood glucose while their fasting blood glucose is normal. It can be speculated that monitoring postprandial TG and RC levels may be more important than their testing in the fasting state in some individuals, just like the importance of postprandial blood glucose detection for diabetes diagnosis and treatment.

Consistent with previous findings, BMI was considered as an independent predictor of elevated TG levels (19). In this present study, the NoTG group was also found to have more individuals with higher BMI. These data demonstrated that elevated TG levels were significantly associated with overweight and/or obesity (20).

There were several limitations in this study. First, compared with similar clinical studies (21), the sample size of this study is relatively small. Second, the levels of blood lipids, especially TG, in those inpatients may be affected by underlying diseases and/or lipid-lowering drugs. Third, the exact amount of nutrition consumed in the breakfast by the patients was unknown. Compared with the undefined meals, the nutrient content of the standard meals is relatively constant and uniform, which makes it more convincing to compare the postprandial TG levels among individuals after standard meals. However, on the one hand, the scope of application of standard meals is still limited. For example, for patients with diabetes, the nutrition of standard meals may be excessive, and the type of nutrients may not be suitable. Therefore, standard meals may not be “standard,” and diverse kinds of nutritious meals should be formulated considering the specific energy needs of different disease populations. On the other hand, in the real world, undefined meals are more accessible, especially for outpatients. Additionally, emerging evidence showed that the dining places may also have some influence on postprandial lipid levels (22), and undefined meals are usually obtained at home, which seems to have little effect on postprandial lipid levels. Taken together, in an ideal state or among healthy people, the postprandial lipid levels after a standard meal are more convincing and suitable. While in the real world, due to their availability, the advantages of dining places and the concern for patients with diverse diseases, undefined meals seem to be more recommended. However, further studies on the sensitivity and specificity of the diagnosis of dyslipidemia through comparison between standardized and daily meals are still needed.

## Conclusion

Taken together, we for the first time determined TG 1.56 mmol/L as the postprandial cut-off value corresponding to fasting TG 1.2 mmol/L in Chinese subjects. This could make it more convenient to determine whether TG is optimal or not in the fasting or postprandial state.

## Data availability statement

The original contributions presented in this study are included in this article/Supplementary material, further inquiries can be directed to the corresponding authors.

## Ethics statement

The studies involving human participants were reviewed and approved by the Ethics Committee of the Second Xiangya Hospital of Central South University and informed consent was obtained from all participants. The patients/participants provided their written informed consent to participate in this study.

## Author contributions

LL and TW were the primary investigators and designers of this study. YX, LG, HC, JX, PQ, LZ, YT, MZ, and TW participated in the design of this study. All authors contributed to the article and approved the submitted version, accepted responsibility for the entire content of this manuscript, and approved its submission.

## Supplementary material

Supplementary Figure 1 Correlation analysis between the levels of triglyceride (TG) and remnant cholesterol (RC) at different time-points. **(A)** Correlation between fasting TG (fTG) and pTG2h levels. **(B)** Correlation between pTG2h and pRC2h. **(C)** Correlation between fTG and pTG 2 and 4h levels. **(D)** Correlation between levels of pTG 2 and 4h and pRC 2 and 4h.

Supplementary Figure 2 Comparison of percentage of patients with non-optimal triglyceride (NoTG) in the fasting state and postprandial state 2 or 4h according to fasting cut-off value. **P* < 0.05 when compared with fasting state, ^#^*P* < 0.05 when compared with postprandial state at 2h.
