# Supplementary figures and images for: Determination of the postprandial cut-off value of triglyceride after a daily meal corresponding to fasting optimal triglyceride level in Chinese subjects
Source: Front Nutr. 2023 Mar 2;10:1037270. doi: 10.3389/fnut.2023.1037270 (PMC10017968; doi:10.3389/fnut.2023.1037270)

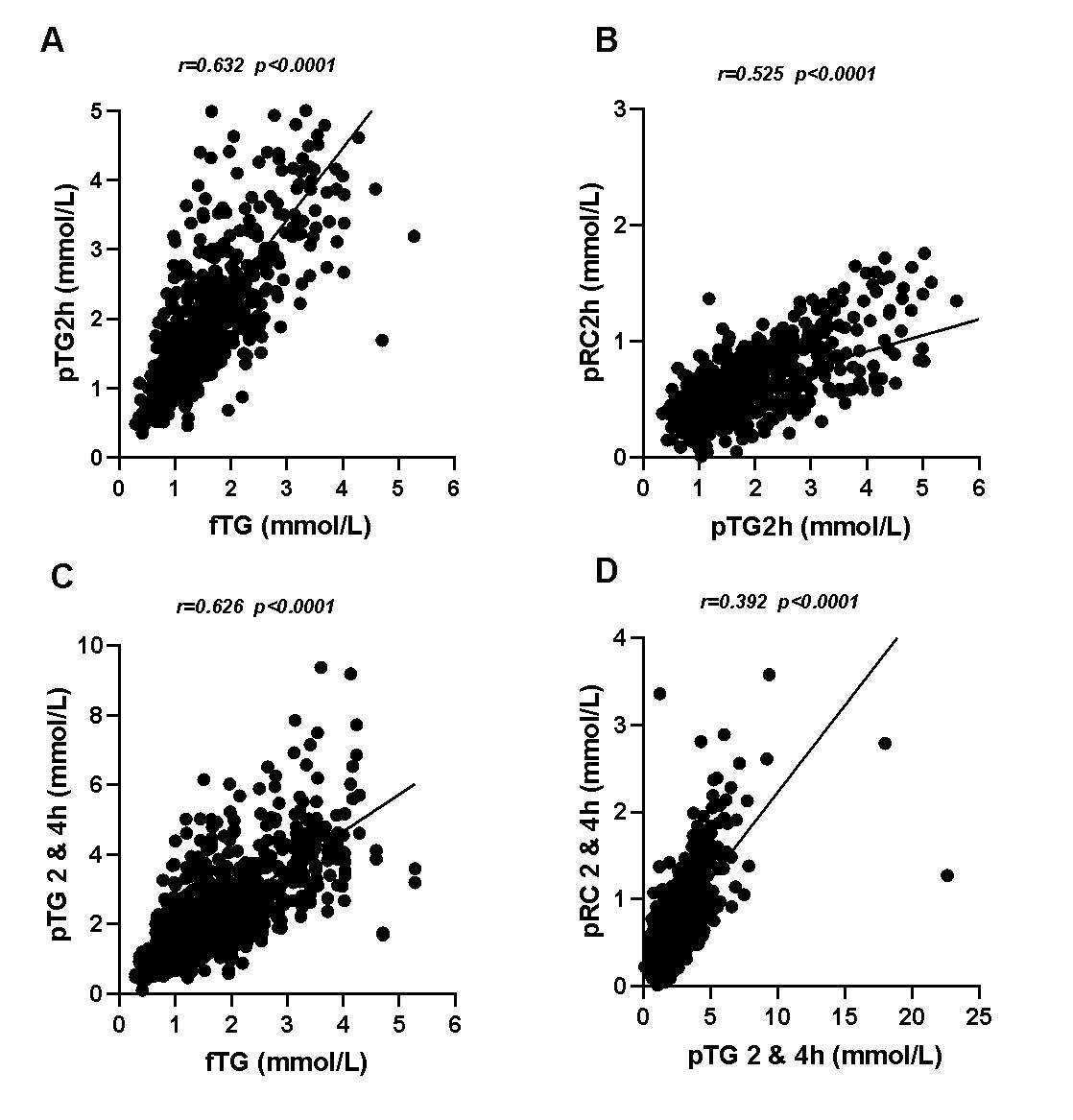

Supplement: Supplementary Figure 1 — Correlation analysis between the levels of triglyceride (TG) and remnant cholesterol (RC) at different time-points. (A) Correlation between fasting TG (fTG) and pTG2h levels. (B) Correlation between pTG2h and pRC2h. (C) Correlation between fTG and pTG 2 and 4h levels. (D) Correlation between levels of pTG 2 and 4h and pRC 2 and 4h. [file Image_1.JPEG]

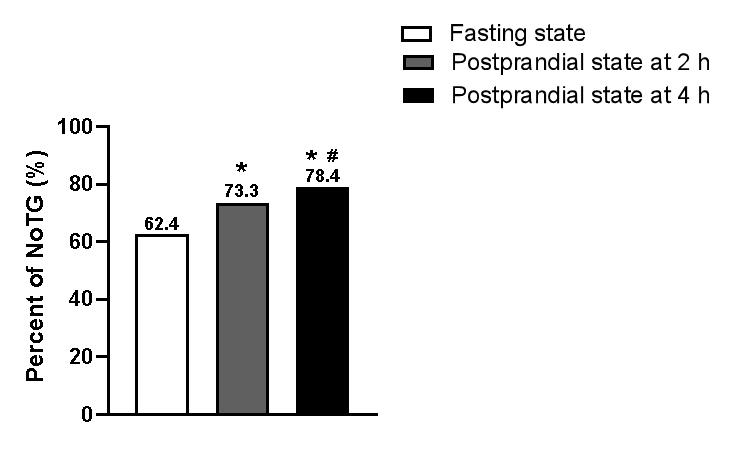

Supplement: Supplementary Figure 2 — Comparison of percentage of patients with non-optimal triglyceride (NoTG) in the fasting state and postprandial state 2 or 4h according to fasting cut-off value. *P < 0.05 when compared with fasting state, #P < 0.05 when compared with postprandial state at 2h. [file Image_2.JPEG]
